# CRSwNP in the biologics era: evaluating patient-reported quality of life outcomes

**DOI:** 10.3389/falgy.2026.1846744

**Published:** 2026-05-28

**Authors:** Tianhui Kang, Hong Qiao, Yang Zha, Xiaowei Wang, Guanshen Ma, Wei Lv, Surita Aodeng

**Affiliations:** Department of Otolaryngology, Peking Union Medical College Hospital, Chinese Academy of Medical Sciences and Peking Union Medical College, Beijing, China

**Keywords:** biologic therapy, chronic rhinosinusitis with nasal polyps, patient-reported outcome measures, quality of life, type 2 inflammation

## Abstract

Chronic rhinosinusitis with nasal polyps (CRSwNP) is a prevalent type 2 inflammatory disorder that substantially impairs patient quality of life (QoL) through cardinal symptoms of nasal obstruction, olfactory dysfunction, and sleep disturbance, further compounded by frequent comorbidities including asthma and non-steroidal anti-inflammatory drug-exacerbated respiratory disease (NERD). While conventional therapies with intranasal corticosteroids, systemic corticosteroids, and endoscopic sinus surgery remain foundational, their limitations in achieving durable disease control have prompted the emergence of biologic agents targeting key type 2 inflammatory pathways. This narrative review synthesizes evidence from a targeted literature search including randomized controlled trials and complementary real-world studies to evaluate the impact of biologic therapies on patient-reported QoL outcomes in severe CRSwNP. Biologic agents targeting key mediators of type 2 inflammation have consistently demonstrated clinically meaningful improvements in disease-specific QoL, as measured by the Sinonasal Outcome Test-22 (SNOT-22), across nasal, sleep, olfactory, and emotional domains, with consistent benefits regardless of comorbidities or prior surgery. Real-world evidence corroborates trial findings, demonstrating sustained QoL benefits and reduced dependence on systemic corticosteroids and revision surgery. The integration of patient-reported outcome measures into therapeutic decision-making bridges the gap between objective clinical metrics and lived disease burden, informing patient-centered care models. Biologic therapies have redefined success in CRSwNP management by consistently delivering multidimensional, durable improvements in patient-reported QoL, head-to-head comparisons and optimized treatment sequencing warrant further research.

## 1 Introduction

Chronic rhinosinusitis with nasal polyps (CRSwNP) is a prevalent chronic inflammatory airway disorder, affecting up to 12% of the global population (1, 2). It is characterized by persistent sinonasal mucosa inflammation, with type 2 inflammation being the predominant endotype (3, 4). Type 2 inflammation is primarily driven by key cytokines including interleukin (IL)-4, IL-5, and IL-13, which promote eosinophilic infiltration, elevated immunoglobulin E (IgE) synthesis, and subsequent mucosal injury (3, 5). Consequently, these inflammatory mediators have become critical therapeutic targets in the management of CRSwNP (2, 6).

CRSwNP substantially impairs patients' quality of life (QoL), primarily through symptoms such as nasal obstruction, hyposmia or anosmia, and facial pain (7). The burden is amplified by frequent comorbidities, particularly allergic rhinitis, asthma and non-steroidal anti-inflammatory drug-exacerbated respiratory disease (NERD), which complicate treatment and worsen outcomes (3, 5, 8–12).

The therapeutic landscape for CRSwNP has recently undergone a major paradigm shift. While corticosteroids and endoscopic sinus surgery have traditionally constituted the mainstay of treatment, the emergence of biologic therapies has expanded options for patients with inadequate responses to standard care (13, 14). By targeting key type 2 inflammatory mediators such as IL-5 and IL-4 receptor alpha (IL-4R*α*), these biologic agents offer a precision medicine approach (15–17). Clinical evidence has demonstrated their efficacy in reducing polyp burden, decreasing corticosteroid dependency, and, in selected cases of severe refractory disease, obviating the need for surgical intervention (18, 19).

Traditional objective metrics like polyp scores do not fully capture the patient's lived experience. This narrative mini review therefore evaluates the impact of biologic therapies on quality of life (QoL) in CRSwNP patients, with a focus on patient-reported outcome measures (PROMs) including the Sinonasal Outcome Test-22 (SNOT-22) (5, 20). A targeted literature search was conducted in PubMed, Embase, and Web of Science up to March 2026, using key terms related to CRSwNP, biologic therapies, QoL, and PROMs. Recent randomized controlled trials, real-world studies, and key guidelines relevant to biologic effects on patient-reported QoL were selected. Studies were excluded if they lacked QoL-related outcomes, were irrelevant to biologic therapy for CRSwNP, were duplicate publications, or were of low methodological quality. Only high-impact, clinically relevant studies were retained to ensure focus and conciseness. This article aims to provide an updated overview of how biologic therapies may contribute to improved outcomes in CRSwNP management, and to support evidence-based decision-making in the era of personalized medicine.

## 2 The impact of CRSwNP on quality of life

### 2.1 Symptom burden and functional impairment

CRSwNP imposes a substantial and multidimensional burden on QoL, with nasal congestion, rhinorrhea, and facial pain consistently associated with elevated SNOT-22 scores and impaired daily functioning (12, 21). Olfactory dysfunction, a hallmark of CRSwNP, compromises food enjoyment, personal safety, and social interactions. It is also strongly associated with depression and overall disease severity (10, 22). Sleep disturbance affects over three-quarters of patients, reflecting inflammatory activity beyond mechanical obstruction and contributing to fatigue and reduced productivity (23, 24). From a psychological perspective, patients frequently report embarrassment, frustration, anxiety, and depressed mood, underscoring the considerable mental health burden of inadequately controlled disease (12, 21).

### 2.2 Impact of comorbid conditions on disease burden

Comorbidities, particularly allergic rhinitis, asthma and NERD, play a critical role in amplifying QoL impairment among patients with CRSwNP. Allergic rhinitis is one of the most prevalent comorbidities in CRSwNP, with a reported comorbidity rate as high as 50%–70%, and the two conditions share overlapping type 2 inflammatory mechanisms and risk factors (12, 25). A real-world study of 683 patients with moderate-to-severe CRSwNP found that 69% had at least one type 2 comorbidity, with allergic rhinitis being the most common (26). Concurrent allergic rhinitis exacerbates sinonasal symptoms including nasal obstruction, rhinorrhea, and olfactory disturbance, further reducing health-related quality of life and increasing the risk of CRSwNP exacerbations and treatment resistance (25).

Asthma is observed in 40%–67% of CRSwNP patients and is strongly correlated with heightened disease severity, more frequent exacerbations, and an increased need for surgical interventions (27, 28). The co-occurrence of asthma and CRSwNP is particularly detrimental, as both conditions share common type 2 inflammatory pathways, leading to a compounded negative impact on patients' health status (29, 30). Patients with concomitant asthma experience poorer disease control and more frequent exacerbations, factors that significantly diminish their overall QoL (31).

NERD represents a particularly severe and clinically challenging subtype of CRSwNP. Affected individuals experience pronounced olfactory dysfunction and persistent sinonasal inflammation, which further compromise QoL and complicate therapeutic management (32). These patients frequently demonstrate resistance to conventional first-line therapies, necessitating repeated surgical procedures and advanced biological treatments (33, 34). The complexities of managing NERD increase healthcare utilization and treatment duration, imposing substantial physical and psychological strain (31, 35). Emerging evidence suggests that biologic therapies targeting type 2 inflammation may offer symptomatic relief in this challenging patient population, potentially improving both disease control and QoL outcomes (36, 37).

## 3 Patient-reported outcome measures in CRSwNP

### 3.1 Disease-specific instruments

Patient-reported outcome measures are essential tools for assessing the QoL and symptom burden in patients with CRSwNP. Among disease-specific PROMs, the SNOT-22, Chronic Rhinosinusitis-Patient Reported Outcome (CRS-PRO), and Total Nasal Symptom Score (TNSS) are the most frequently employed in both clinical practice and research settings, enabling patients to directly report their symptoms, offering invaluable insights into their lived experience of the disease (25, 38). The SNOT-22 is the most widely utilized, assessing symptom severity across five domains: rhinologic symptoms, extra-nasal rhinologic symptoms, ear and facial symptoms, psychological dysfunction, and sleep disturbance (39). It is frequently employed to quantify disease severity and evaluate therapeutic outcomes (25). A decrease of approximately 9 points or more in the total SNOT-22 score is widely accepted as the minimal clinically important difference (MCID), a threshold used throughout this review to denote meaningful improvement (40, 41). In contrast, the CRS-PRO is a concise instrument specifically developed for chronic rhinosinusitis populations. It encompasses three core factors: rhinologic symptoms, psychosocial impact, and facial discomfort. This tool has been linguistically validated in multiple languages and has demonstrated excellent reliability and responsiveness (42). Meanwhile, the TNSS focuses specifically on cardinal nasal symptoms, including congestion, rhinorrhea, sneezing, and nasal itching. It has been integral in studies evaluating biologic efficacy for CRSwNP, such as omalizumab (25).

### 3.2 Complementary role of generic health instruments

In addition to disease-specific PROMs, general health instruments play a vital role in providing a broader assessment of the overall QoL in CRSwNP patients. The EuroQol 5-Dimension (EQ-5D) captures general health status across five dimensions, namely mobility, self-care, usual activities, pain or discomfort, and anxiety or depression, and includes a visual analog scale (VAS) for self-rated health, offering a comprehensive evaluation of QoL that extends beyond the specific symptoms of the disease (43, 44). Furthermore, the Medical Outcomes Study Short Form-36 (SF-36) serves as another critical tool for assessing broader health-related outcomes across eight domains, aggregated into physical and mental component summary scores, although it has demonstrated limited long-term responsiveness in some studies (45).

These general health measures complement disease-specific PROMs like the SNOT-22, thereby providing a more holistic understanding of patient well-being (38). Integrating both instrument types allows comprehensive monitoring of disease burden and intervention effectiveness, facilitating improved patient management (25).

## 4 Quality of life outcomes with conventional therapies

### 4.1 Medical therapy: intranasal and systemic corticosteroids

The conventional management for CRSwNP generally encompasses intranasal corticosteroids (INCS), systemic corticosteroids (SCS), and endoscopic sinus surgery (ESS). INCS constitute the cornerstone of first-line pharmacotherapy, demonstrating substantial efficacy in alleviating nasal congestion, reducing polyp size, and mitigating the need for surgical intervention or additional systemic corticosteroid courses (46). Their favorable safety profile underscores their role as a safe and effective option for long-term management (46, 47). In contrast, SCS are typically reserved for short-term management of acute exacerbations, providing rapid relief from severe symptoms including olfactory dysfunction and significant polyp burden (48, 49). However, the therapeutic benefits are transient and do not persist following treatment cessation (48). Moreover, protracted or repeated administration is associated with substantial cumulative risks, including avascular necrosis, pneumonia, obesity, sleep apnea, diabetes, and hypertension (50). While SCS are commonly administered preoperatively to optimize surgical field visualization, their extended use remains constrained by this unfavorable risk-benefit profile (48, 51).

### 4.2 Surgical therapy: endoscopic sinus surgery and extended approaches

Endoscopic sinus surgery represents an essential therapeutic modality for patients with CRSwNP inadequately controlled by medical therapy alone, with established efficacy in improving QoL outcomes (52). Among the various surgical approaches, Functional Endoscopic Sinus Surgery (FESS) has demonstrated substantial improvements in health-related quality of life (HRQoL), particularly in CRSwNP patients. Notably, patients presenting with poorer preoperative HRQoL scores exhibit the most significant postoperative improvements, highlighting the critical role of patient selection in achieving optimal treatment outcomes (53, 54). Additionally, extended surgical techniques such as Extended Frontal Sinusotomy (EFS), particularly Draf IIb or Draf III procedures, have shown significant improvements in QoL as measured by the SNOT-22 questionnaire (8). Patients' systemic corticosteroid usage has significantly decreased, indicating improved disease control post-surgery (8).

## 5 Quality of life in the biologics era

### 5.1 Dupilumab (anti-IL-4R*α*)

The pivotal SINUS-24 and SINUS-52 trials evaluated dupilumab, a fully human monoclonal antibody blocking IL-4R*α* (17). Treatment led to significant reductions in SNOT-22 total and domain scores, alongside improvements in EQ-VAS, reflecting benefits in both disease-specific and general health status at Week 24, with sustained gains through Week 52 (55, 56). QoL improvements were observed across nasal, sleep, functional, and emotional components (57, 58). Benefits were consistent regardless of comorbid asthma, NERD, prior sinus surgery, or baseline biomarker levels, underscoring the broad applicability and durability of dupilumab's HRQoL effects (30, 59).

### 5.2 Omalizumab (anti-IgE)

Data from the POLYP-1 and POLYP-2 trials provide robust evidence for omalizumab, a recombinant humanized monoclonal antibody targeting IgE (60). Treatment resulted in clinically meaningful and statistically significant reductions in SNOT-22 total scores, indicative of substantial improvements in daily functioning and well-being (25, 60). These benefits spanned multiple symptom domains, including nasal obstruction, rhinorrhea, and olfactory dysfunction, highlighting broad symptom relief (60, 61). Subsequent analyses confirmed consistent HRQoL improvements irrespective of allergic status or comorbid asthma (62, 63). Notably, omalizumab also improved sleep-related QoL, with parallel reductions in SNOT-22 sleep domain scores, translating into reduced fatigue, enhanced productivity, and improved perceived health status (60, 64). As an anti-IgE agent, omalizumab also provides consistent benefits for comorbid allergic rhinitis symptoms, further contributing to improved overall quality of life in allergic CRSwNP patients (25, 60, 63).

### 5.3 Mepolizumab (anti–IL-5)

Targeting the eosinophilic endotype, mepolizumab was assessed in the SYNAPSE trial, reinforcing the role of IL-5-driven pathways in CRSwNP morbidity (65). Compared with placebo, mepolizumab led to substantial reductions in SNOT-22 total scores and VAS measures of individual symptoms, reflecting broad amelioration of disease-related burden (65, 66). Patients receiving mepolizumab were more likely to achieve meaningful within-patient improvements across multiple symptom domains, including nasal obstruction and overall functional impairment (65, 67). These QoL benefits were consistent across key clinical subgroups, including patients with comorbid asthma or NERD (68). Furthermore, mepolizumab significantly improved olfactory-related QoL, particularly among patients with fewer or more recent prior sinus surgeries (69).

### 5.4 Benralizumab (anti-IL-5R*α*)

The OSTRO trial investigated benralizumab and its effects on both objective and patient-reported outcomes in CRSwNP (70). Treatment with benralizumab resulted in reductions in SNOT-22 total scores, encompassing nasal symptoms, sleep disturbance, and daily functioning (70, 71). These benefits were accompanied by significant reductions in nasal polyp score and nasal congestion score at Week 40, indicating improved objective disease control (70, 71). Although olfactory outcomes were generally more modest, nominal or statistically significant improvements in smell perception were reported in OSTRO and complementary analyses, suggesting potential olfactory-related QoL benefits in selected patient populations (70, 72). Subgroup analyses from the OSTRO trial further suggested that baseline characteristics such as comorbid asthma, number of prior sinus surgeries, and baseline blood eosinophil count may influence treatment response, including olfactory outcomes (70, 73). However, it is critical to note that despite these promising efficacy signals, the supplemental Biologics License Application for benralizumab in CRSwNP has not been approved by the US Food and Drug Administration (FDA). Consequently, benralizumab is currently not approved for the routine treatment of CRSwNP in major markets like the United States.

### 5.5 Depemokimab (long-acting anti-IL-5)

Depemokimab, a long-acting anti-IL-5 monoclonal antibody with a six-month dosing interval, was evaluated in the ANCHOR-1 and ANCHOR-2 trials (74). Reductions in SNOT-22 scores reflect decreased disease burden, consistent with QoL benefits observed with other IL-5–targeting biologics and supporting the central role of eosinophil suppression in symptom control (66, 74). By alleviating key symptoms such as nasal congestion and olfactory dysfunction, depemokimab may improve sleep quality and daily functioning (75). Additionally, reductions in type 2 inflammation may enhance control of comorbid asthma and NERD, indirectly contributing to overall QoL improvements (74, 76). The extended dosing interval of approximately six months may reduce treatment burden, supporting long-term adherence and sustained QoL gains (73). While highly promising, the regulatory status of depemokimab for CRSwNP is still evolving. Based on the ANCHOR trials, it has received approval from the UK's Medicines and Healthcare products Regulatory Agency (MHRA). However, its CRSwNP indication is not yet approved by the US FDA. In China, depemokimab has been approved by the National Medical Products Administration (NMPA) for the treatment of adults with CRSwNP inadequately controlled by systemic corticosteroids and/or surgery, as the first and only ultra-long-acting biologic for this indication in China.

### 5.6 Tezepelumab (anti-TSLP)

Clinical data from the WAYPOINT and NAVIGATOR studies indicate that tezepelumab targeting thymic stromal lymphopoietin (TSLP) confers substantial and clinically meaningful improvements in QoL among patients with CRSwNP (77, 78). In WAYPOINT, treatment with tezepelumab resulted in robust reductions in SNOT-22 scores compared with placebo at Week 52, reflecting marked symptom relief and reduced disease burden (77). *post hoc* analyses of the NAVIGATOR trial in patients with severe uncontrolled asthma and comorbid CRSwNP corroborate these findings, showing significantly greater improvements in total SNOT-22 scores vs. placebo (79). Systematic reviews and network meta-analyses further ranked tezepelumab among the most effective biologics for SNOT-22 improvement, second only to dupilumab, underscoring its strong efficacy profile (13). Collectively, these findings support tezepelumab as an effective option for improving HRQoL in biologics-eligible CRSwNP populations.

### 5.7 Stapokibart (anti-IL-4R*α*)

The CROWNS-2 trial examined stapokibart, a novel anti-IL-4R*α* monoclonal antibody, demonstrating statistically significant and clinically meaningful effects, with particularly notable benefits in olfactory function and comorbid asthma control (19). Treatment resulted in a least-squares mean reduction in SNOT-22 score compared with placebo, exceeding the minimal clinically important difference (19, 80). These QoL benefits were accompanied by substantial reductions in nasal polyp score and nasal congestion score, reflecting improved disease control and daily functioning (80). Stapokibart also significantly enhanced olfactory function, as evidenced by improvements in T&T olfactometry scores (19). Notably, among eosinophilic CRSwNP patients with comorbid asthma, stapokibart concurrently improved asthma control and pulmonary function indices, highlighting integrated benefits across upper and lower airway disease and their combined impact on overall QoL (19, 80).

**Figure 1:**
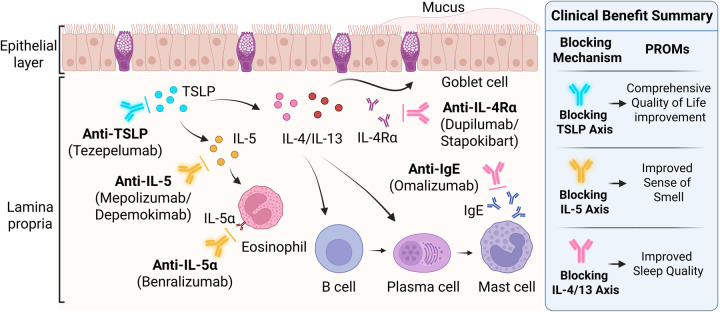
Chronic rhinosinusitis with nasal polyps targeted therapy: From molecular mechanisms to quality of life. This figure summarizes the molecular pathways targeted by biologic agents for Chronic Rhinosinusitis with Nasal Polyps (CRSwNP), including inhibitors of the TSLP, IL-5, and IL-4/IL-13 axes. It illustrates how blockade of key type 2 inflammatory cytokines translates into measurable improvements in patient-reported outcome measures (PROMs), delivering multidimensional quality of life (QoL) benefits for patients. (Created with BioRender.com). CRSwNP, chronic rhinosinusitis with nasal polyps; IgE, immunoglobulin E; IL, interleukin; IL-4R*α*, interleukin-4 receptor *α*; TSLP, thymic stromal lymphopoietin; PROMs, patient-reported outcome measures; QoL, quality of life.

Figure 1 presents a mechanistic overview of targeted biologic therapies for severe CRSwNP, bridging molecular-level blockade of type 2 inflammatory pathways with the corresponding patient-reported quality of life improvements observed in clinical trials (Figure 1). To facilitate direct comparison, Table 1 summarizes key clinical outcomes from the pivotal phase III trials discussed, including patient-reported and objective measures (Table 1).

**Table 1:** Clinical outcomes of biologic treatment and impact on quality of life and nasal polyp score for patients with chronic rhinosinusitis with nasal polyps.

Biologic	Treatment	Comparator treatment	Study type	Chronic rhinosinusitis with nasal polyps patient population (enrolled/randomized)	Study duration	Key PROM used	Outcomes (QoL change)	Outcomes (objective change: NPS change)	Regulatory Status (CRSwNP Indication)	Reference
Dupilumab	300 mg subcutaneously every 2 weeks plus mometasone (200 μg/day)	PBO plus mometasone (200 μg/day)	RCT	*n* = 448 (267 with asthma, 120 with NERD) in SINUS 52	4-week run-in period, followed by 52-week treatment, followed by 12-week follow-up	SNOT-22	SNOT-22, PBO versus Dupilumab, –27.77 versus –10.40 (*P* < 0.0001) in SINUS-52	NPS, PBO versus Dupilumab, –1.71 versus 0.10 (*P* < 0.0001) in SINUS-52	FDA/EMA: Approved	(15)
Omalizumab	75–600 mg subcutaneously every 2–4 weeks plus mometasone (200 μg/day or twice daily)	PBO plus mometasone (200 μg/day or twice daily)	RCT	*n* = 138 (74 with asthma, 27 with NERD) in POLYP 1	24 weeks, followed by 4-week follow-up	SNOT-22	SNOT-22, Omalizumab versus PBO, –24.7 versus –8.6 (*P* < 0.0001) in POLYP 1 and –21.6 versus –6.6 (*P* < 0.0001) in POLYP 2	NPS, Omalizumab versus PBO, –1.08 versus 0.06 (*P* < .0001) in POLYP 1 and –0.90 versus –0.31 (*P* = 0.0140) in POLYP 2	FDA/EMA: Approved	(59)
				*n* = 127 (77 with asthma, 45 with NERD) in POLYP 2						
Mepolizumab	100 mg every 4 weeks plus mometasone (200 μg/day), saline nasal irrigation, systemic corticosteroids, or antibiotics (as required)	PBO plus mometasone (200 μg/day), saline nasal irrigation, systemic corticosteroids, or antibiotics (as required)	RCT	*n* = 407 (289 with asthma, 108 with NERD)	52 weeks	VAS (nasal obstruction), SNOT-22	Nasal obstruction VAS score, Mepolizumab versus PBO, –4.2 versus –2.5 (*P* < 0.0001) SNOT-22, Mepolizumab versus PBO, –29.4 versus –15.7 (*P* = 0.0032)	NPS, Mepolizumab versus PBO, –0.9 versus –0.1 (*P* < 0.0001)	FDA/EMA: Approved	(64)
Benralizumab	30 mg subcutaneously every 4 weeks for the first 3 doses, then every 8 weeks, plus mometasone (400 μg/day)	PBO plus mometasone (400 μg/day)	RCT	*n* = 410 (278 with asthma, 121 with NERD)	5-week run-in period; primary efficacy assessment at week 40; treatment through week 56; extended follow-up to week 80	SNOT-22	SNOT-22 at week 40, Benralizumab versus PBO, –16.23 versus –11.02 (*P* = 0.08)	NPS at week 40, Benralizumab versus PBO, –0.418 versus 0.153 (*P* < 0.001)	FDA/EMA: Not Approved	(69)
							SNOT-22 at week 56, Benralizumab versus PBO, –16.25 versus –8.75 (P = 0.02)	NPS at week 56, Benralizumab versus PBO, –0.361 versus 0.114 (P = 0.005)		
Depemokimab	100 mg subcutaneously every 26 weeks plus mometasone (400 μg/day)	PBO plus mometasone (400 μg/day)	RCT	*n* = 271 (161 with asthma, 43 with NERD) in ANCHOR-1	52 weeks, followed by 4-week follow-up	SNOT-22	SNOT-22, Depemokimab versus PBO, –13.3 versus –6.5 (*P* = 0.113) in ANCHOR-1 and –15.9 versus –6.0 (*P* = 0.015) in ANCHOR-2	NPS, Depemokimab versus PBO, –0.6 versus 0.2 (*P* < 0.001) in ANCHOR-1 and –0.5 versus 0.1 (*P* = 0.004) in ANCHOR-2	MHRA/NMPA: Approved	(73)
				*n* = 257 (131 with asthma, 42 with NERD) in ANCHOR-2						
Tezepelumab	210 mg subcutaneously every 4 weeks plus mometasone (400 μg/day)	PBO plus mometasone (400 μg/day)	RCT	*n* = 408 (248 with asthma, 71 with NERD)	52 weeks, followed by 12 or 24 weeks follow-up	SNOT-22	SNOT-22, Tezepelumab versus PBO, –45.02 versus –17.58 (*P* < 0.001)	NPS, Tezepelumab versus PBO, –2.46 versus –0.38 (*P* < 0.001)	FDA/EMA: Approved	(76)
Stapokibart	300 mg subcutaneously every 2 weeks plus mometasone (200 μg/day)	PBO plus mometasone (200 μg/day)	RCT	*n* = 179 (89 with asthma)	4-week run-in period; 24-week double-blind treatment; 28-week open-label extension with stapokibart; 8-week follow-up	SNOT-22, EQ-5D-5L VAS	SNOT-22, Stapokibart versus PBO, –23.5 versus –11.4 (*P* < 0.001)	NPS, Stapokibart versus PBO, –2.6 versus –0.3 (*P* < 0.001)	NMPA: Approved	(17)
							EQ-5D-5L VAS, Stapokibart versus PBO, 4.2 versus 1.4 (P not reported)			

CRSwNP, chronic rhinosinusitis with nasal polyps; EQ-5D, EuroQol 5-Dimension; EQ-5D-5L, EuroQol5-Dimension5-Level questionnaire; FDA, Food and Drug Administration (USA); EMA, European Medicines Agency; MHRA, Medicines and Healthcare products Regulatory Agency (UK); NERD, non-steroidal anti-inflammatory drug-exacerbated respiratory disease; NMPA, National Medical Products Administration (China); NPS, nasal polyp score; PBO, placebo; PROM, patient reported outcome measure; QoL, quality of life; RCT, randomized controlled trial; SNOT-22, Sinonasal Outcome Test-22; VAS, visual analog scale.

## 6 Real-World evidence on biologic therapy

### 6.1 Effectiveness in routine clinical practice

Real-world studies provide complementary evidence to randomized controlled trials, demonstrating that biologic therapies produce rapid and sustained improvements in QoL among patients with CRSwNP (81). Large observational cohorts and registries report marked reductions in SNOT-22 scores, nasal polyp burden, and symptom severity across heterogeneous populations, including those with multiple comorbidities (82). Despite challenges like inconsistent adherence and limited access, sustained benefits on daily functioning and olfaction have been observed (48). While controlled trials offer robust assessments of efficacy under ideal conditions, real-world studies contribute critical insight into therapeutic effectiveness across broader, more heterogeneous patient populations (81). These studies also highlight real-world barriers, such as inconsistent adherence and limited access to biologics, which may influence long-term QoL outcomes (81), thereby informing clinical decision-making in ways that complement trial-derived evidence (75).

### 6.2 Comparative observations

Real-world evidence further provides critical comparative insights into biologics vs. repeated endoscopic sinus surgery (ESS) for refractory CRSwNP, particularly in patients with high type 2 inflammatory burden and recurrent polyposis (7). Observational cohort studies consistently report substantial reductions in revision surgery rates among patients receiving biologic therapy, accompanied by sustained improvements in HRQoL across multiple domains, including nasal obstruction, olfactory function, sleep quality, and overall disease control (18, 42). Compared with serial surgery, biologics demonstrate favorable safety and incremental QoL gains over time (83). Additional benefits are particularly evident in patients with comorbid allergic rhinitis, asthma or NERD, wherein biologic agents enable concurrent control of both upper and lower airway inflammation, a therapeutic advantage not achievable with surgery alone (7). These real-world observations support biologic therapy as a valuable alternative to repeat surgery in appropriately selected severe, refractory patients.

## 7 Future research in patient-centered outcomes

The rapid expansion of biologic therapies in CRSwNP has underscored the importance of PROMs as central indicators of therapeutic success. The next phase of research must move toward dynamic, patient-centered monitoring frameworks.

### 7.1 Digital symptom tracking and ecological monitoring

Emerging digital health technologies offer opportunities to capture symptom variability in real time. Mobile health applications and wearable-integrated platforms may enable longitudinal tracking of nasal obstruction, olfactory perception, sleep quality, and fatigue, thereby providing ecologically valid representations of disease burden (84). Such tools could complement traditional instruments like the SNOT-22 by capturing fluctuations between clinic visits, enhancing responsiveness assessment, and facilitating earlier intervention in cases of disease relapse.

### 7.2 Remote PROM monitoring and telemedicine integration

The integration of remote PROM collection into routine care pathways represents a critical step toward patient empowerment. Automated digital reporting systems may allow patients to submit SNOT-22, EQ-5D, or symptom-specific scales at predefined intervals, enabling clinicians to monitor treatment response longitudinally without requiring in-person visits (85, 86).

### 7.3 Artificial intelligence–based prediction of QoL response

Advances in predictive analytics and machine learning hold promise for personalized biologic selection (87). Integrating baseline biomarkers, comorbidity profiles, and longitudinal PROM trajectories may enable accurate prediction of individuals most likely to achieve clinically significant QoL improvements, refining treatment strategies and minimizing trial and error (88–90).

## 8 Conclusion

 This review identifies that biologic therapies consistently deliver substantial and durable improvements in disease-specific PROMs, particularly SNOT-22, with parallel gains in olfaction, sleep, and daily functioning across phenotypes and comorbidities. Targeting type 2 inflammation achieves multidimensional HRQoL benefits beyond anatomic polyp reduction, while reducing dependence on systemic corticosteroids and revision surgery, representing a paradigm shift toward precision long-term disease control. Integrating PROMs into therapeutic decision-making helps fill longstanding gaps between objective metrics and lived disease burden, informing patient-centered care models.

Notably, meaningful PROM improvements may persist in some patients after biologic cessation or dose tapering, suggesting that biologics can induce sustained remission of type 2 inflammation in responsive individuals (67, 81, 89).

Nonetheless, important limitations temper current conclusions. Most randomized controlled trials enroll highly selected populations with limited generalizability. Real-world observational studies carry risks of selection bias, confounding, and variable treatment adherence. Meanwhile, heterogeneity exists in PROM instruments and MCID thresholds, baseline disease severity, and comorbidity profiles, with restricted external validity from selected trial populations, and limited reporting of mental health and socioeconomic outcomes. Furthermore, comparative evaluation between different biologic therapies remains insufficient. Network meta-analyses suggest potential efficacy differences: dupilumab ranks highest for SNOT-22 improvement, and stapokibart for nasal polyp reduction (13). However, head-to-head trials remain scarce, and indirect comparisons are limited by across-trial heterogeneity.

Future research should prioritize several key areas. First, longitudinal, head-to-head comparative effectiveness designs integrating mechanistic biomarkers with PROM trajectories are urgently needed to guide biologic selection and sequencing. Second, rigorous cost-effectiveness analyses and health economic analyses are essential to support sustainable access, reimbursement, and equitable implementation in routine clinical practice. Third, long-term safety data extending beyond 52 weeks remain limited and require further investigation in large real-world cohorts. Fourth, research must include underrepresented ethnic and geographic groups to capture culturally grounded QoL constructs. Fifth, standardized core outcome setspaired with digital symptom monitoring may enhance comparability and real-world applicability. Finally, real-world implementation barriers including high treatment costs, variable insurance coverage, and unequal geographic access must be formally addressed to ensure that trial efficacy can be translated into equitable clinical benefit. Advancing these agendas will consolidate the evidence base, refine treatment indications, and ultimately optimize HRQoL outcomes through personalized, data-informed care pathways.
